# On the Blood and Urine

**Published:** 1848-05

**Authors:** 


*On the Blood and Urine.* By John William Griffith, M. D., F. L. S., &c.; C. Owen Rees, M. D., F. R. S., F. G. S., &c.; and Alfred Markwick, M. D., &c. In one volume. 12mo. pp. 113. Lea & Blanchard. Philadelphia: 1848.

Since the increased attention paid, of late, to microscopy and organic chemistry, the study of the blood and urine has become almost a new subject. The attainment of a knowledge of the mere nomenclature of the constituents of the urine, more especially as discovered by the investigations of the organic chemists of the day, is now, indeed, no light labour. Messrs. Lea & Blanchard have then acted *wisely* as regards the interests of the profession, and we hope *profitably* as regards themselves, in republishing in one volume—*first*, the "Practical Manual" of Dr. Griffith, which contains "a description of the general, chemical and microscopical characters of the blood, and secretions of the human body, as well as their components, including both their healthy and diseased states; with the best methods of separating and estimating their ingredients; also a succinct account of the various concretions occasionally found in the body and forming calculi;" *secondly*, the small volume by Dr. G. O. Rees, "on the analysis of the Blood and Urine in health and disease; and on the treatment of Urinary Diseases;" and *thirdly*, the "Guide to the Examination of the Urine in health and disease, for the use of students," by Mr. Markwick. All these are valuable essays, with which it is important that every one should be provided, who is desirous of maintaining himself at all on a level with the existing condition of the important topics on which they treat.

